# Data Collection in Multiple Sclerosis: The MSDS Approach

**DOI:** 10.3389/fneur.2020.00445

**Published:** 2020-06-16

**Authors:** Tjalf Ziemssen, Raimar Kern, Isabel Voigt, Rocco Haase

**Affiliations:** ^1^Center of Clinical Neuroscience, University Hospital Carl Gustav Carus, Dresden, Germany; ^2^MedicalSyn GmbH, Stuttgart, Germany

**Keywords:** multiple sclerosis, documentation, digital patient management, post-authorization safety study, MSDS^3D^

## Abstract

Multiple sclerosis (MS) is a frequent chronic inflammatory disease of the central nervous system that affects patients over decades. As the monitoring and treatment of MS become more personalized and complex, the individual assessment and collection of different parameters ranging from clinical assessments via laboratory and imaging data to patient-reported data become increasingly important for innovative patient management in MS. These aspects predestine electronic data processing for use in MS documentation. Such technologies enable the rapid exchange of health information between patients, practitioners, and caregivers, regardless of time and location. In this perspective paper, we present our digital strategy from Dresden, where we are developing the Multiple Sclerosis Documentation System (MSDS) into an eHealth platform that can be used for multiple purposes. Various use cases are presented that implement this software platform and offer an important perspective for the innovative digital patient management in the future. A holistic patient management of the MS, electronically supported by clinical pathways, will have an important impact on other areas of patient care, such as neurorehabilitation.

## Introduction

The low average age at diagnosis and an only slightly reduced life expectancy make multiple sclerosis (MS) a long-term disease that is relevant to patients for decades (1, 2). At the same time, the high inter- and intra-individual variability in the course of the disease constantly leads to new treatment situations (3). As a result, numerous disease data with information about complaints, symptoms, as well as diagnostic and therapeutic measures accumulate within the framework of medical and therapeutic care (4).

Today, certain therapeutic options are linked to the presence of certain disease characteristics (5). When prescribing specific therapies, the effectiveness must be documented individually for each patient. The differentiation between responders and non-responders of immunomodulatory therapies is not conceivable without efficient specific documentation (6, 7). When the documentation of psychological symptoms and other medical disciplines are added, the necessity for a complex course documentation becomes clear (8, 9). In addition, a large number of healthcare institutions depend on a timely and holistic exchange of information between the partners involved (10, 11).

### Patient Documentation

Electronic patient data management represents a suitable implementation for the MS progress documentation of all the points mentioned. Linkable database systems allow individual courses to be displayed in a standardized way over many years, and the data generated can be stored in a readable, transparent, and quickly retrievable form (12, 13). Automated calculations lower the threshold for the systematic application of established scales such as Expanded Disability Status Scale (EDSS) or Multiple Sclerosis Functional Composite (MSFC), which are indispensable for the quantification of neurological deficits (14–19). In addition to the standard instruments, patient-reported outcomes (PROs) increasingly complete the holistic assessment of the disease course (20, 21). The regular use of scales is now a prerequisite in expert recommendations regarding MS therapy. Patient-specific documentation and management is becoming more and more important in the growing field of neurorehabilitation in MS (22). Of particular importance is how complex and individualized neurorehabilitation is designed. Common approaches to neurorehabilitation include the treatment of individual symptomatic impairments, often using motor training approaches (23, 24). Because of the wide range of symptoms and disabilities in MS, single symptomatic interventions can only be seen as part of the rehabilitation program. Comprehensive information and education of patients and relatives and other social and environmental factors are equally important (25). The more stakeholders are involved and the more information is collected and processed, the more complex and costly the processes of neurorehabilitation become, which leads to the necessity of a measurable efficiency of rehabilitation.

Due to the large amount of data to be processed, the large number of communicating persons, and the demands of the healthcare system, all these points predestine electronic data processing for use in the holistic documentation and management of progression in MS (26–28).

### eHealth for Documentation of Patient Data

The coordinated exchange of health-related information is associated with numerous promising opportunities for daily care in clinics and practices supporting decision-making and the treatment process as a whole (10, 29). Technologies such as an electronic medical record (EMR) enable the rapid exchange of health information between patients, practitioners, and caregivers, regardless of time and location, with the EMR mainly exchanging data between health professionals in single entities of the health system (4, 22, 30–33). Today, every part of the treatment process—from diagnosis, treatment selection, and application to patient education and long-term care, including drug treatment and rehabilitation—can be complemented by a quality-assured implementation of information technologies in healthcare (“eHealth”), which also takes into account data security standards and concerns (4, 34, 35).

Such eHealth services are generally considered useful for physicians and nurses in neurological practices to improve clinical documentation, data collection, and diagnosis of specific MS symptoms, doctor–patient communication, and patient education (33). Practices specialized in MS have an increased need for eHealth services to document interventional and non-interventional drug treatment and rehabilitation studies (36).

Despite the many arguments for detailed electronic documentation of people with MS (pwMS), implementation in clinical practice is difficult and has not yet been standardized. The most significant reason for the lack of acceptance and active use of electronic documentation services is the additional time required. Due to the problematic reimbursement situation for physicians, the additional time for detailed documentation is often lacking, especially since there are no comprehensive initiatives by the funding agencies for this problem, which can be aggravated by non-synergetic double documentation tasks resulting from incompatible data platforms. The interoperability of health data between hospital information systems, documentation systems of physician networks or cooperation projects, study-related platforms, and register databases is often severely limited. Various electronic documentation systems developed by the pharmaceutical industry were not followed up after more or less lengthy pilot phases. Overall, it became apparent how problematic a documentation platform dependent on a single pharmaceutical manufacturer can be.

Cross-project documentation systems or systems that are not limited to a single purpose increase the value and service life of health data. Recent advances in the diagnosis and treatment of MS require far-reaching policy changes in clinical reality in order to develop a holistic and efficient approach to MS management (37). In an ideal scenario of well-connected healthcare providers, the EMR serves as a central source of health information by aggregating multi-modular information from different domains and making it accessible according to the needs of all users, not only in-house healthcare professionals. Due to the heterogeneity of MS, it is of great importance to establish reliable and valid measuring instruments to capture disease-relevant characteristics from the patient's point of view in addition to clinical and imaging procedures (3).

### Integration of the Patient's Perspective

Factors reported by patients themselves such as symptoms, health status, health-related quality of life, but also adherence to and satisfaction with treatment, as well as treatment outcomes, are increasingly becoming the focus of attention. PROs are collected using standardized questionnaires and provide valuable information on the effectiveness of interventions and therapies (20, 38–41). The patients' symptoms and physical impairments remain unexplored by the healthcare providers, especially in the intervals between clinic visits (42–45). In addition, pwMS are often affected by varying degrees of cognitive impairment and may forget what they felt a week or two before planned visits (46–48). One possible solution to this problem is for patients to answer questions about their symptoms electronically, either via Internet or through their app-based electronic devices such as smartphones or tablets (49–51). As we have analyzed, patients are happy to use digital instruments to document their disease status (11, 52). Their responses could then be transferred to the health record and various doctors could receive automated notifications of alarming symptoms, which enables the step from data collection to electronically assisted disease management.

Unlike paper-based documentation with its limitations (missing, ambiguous, or contradictory data), electronic documentation with tablets or smartphones can eliminate these problems (11, 51, 53, 54). This enables a faster and more efficient collection of information, offers high security in data storage, and is environmentally friendly.

In order to enable documentation across cases and institutions, all findings, diagnoses, treatment measures, and reports in the future will be stored in an EMR that must also be accessible and usable by the patient. Such a patient record is the starting point for a digitally supported patient management. It enables the physician to quickly gain an overview of all important data as well as the course of the disease and to offer a personalized treatment to the patient in a process of shared decision-making based on shared information. For example, prescribed medication can be read or a comprehensive clinical picture can be created. The issuing of electronic prescriptions, referrals, or doctor's letters can also contribute to more efficient and cost-effective healthcare. Medical care that is specially tailored to the patient improves the course of the disease by reducing side effects to a minimum. In addition, the acceptance of the medication is increased, which in turn improves the effect of the medication (55–58).

## The MSDS Approach

### From MSDS Clinic to MSDS^3D^

The Multiple Sclerosis Documentation System (MSDS) with its clinical focus was developed in Dresden, Germany. It has established itself as an input platform and is constantly being further developed as a desktop version and for web browsers (24, 59, 60). The first MSDS version (*MSDS Clinic*) was specially designed for MS outpatient departments at universities in 1999 for the structured collection of clinical data on the pathology of MS as well as for the writing of letters to physicians. For the first time, it allowed several users to access the database at the same time. MSDS found a growing number of users in Germany and was used in the MS Registry pilot project. In its early EMR-like version, MSDS allowed the user to enter patient data, clinical history and clinical examination data as well as results and treatment details. For the first time, it was possible to graphically display the course of an individual patient and create medical reports (60).

*MSDS Practice* is a modified version of the above mentioned clinical MSDS version designed specifically for neurological outpatient practices. In contrast to *MSDS Clinic, MSDS Practice* addresses the special requirements of neurological practices through a reduced scope of documentation and a simplified user interface, and it combines a transparent presentation of the course of disease with diagnostic and therapeutic decisions in everyday practice (59).

In view of the increasingly complex therapies, the eHealth project group at Dresden University Hospital developed the multidimensional patient management system *MSDS*^3*D*^ in cooperation with MedicalSyn GmbH in 2014. As a further development of the *MSDS Clinic, MSDS*^3*D*^ is designed to support physicians in performing more complex processes (e.g., treatment management) and integrates patient, nurse, and physician into these processes. Especially in the case of complex long-term diseases such as MS, those involved in the treatment process want a special, intelligent management system that goes beyond pure documentation (61). In addition, the system can be used not only to enter and interpret patient data, but also as an interactive system to provide information to the patient. Interaction with patients takes place either via multi-touch systems as an interactive patient terminal or via mobile devices such as the patient's smartphone. With the development of *MSDS*^3*D*^, the step from pure patient documentation to an adaptive patient management system for MS was thus completed (4, 24).

### Patient Data in MSDS^3D^

*MSDS*^3*D*^ can be used to conduct the preliminary and accompanying examinations necessary for the application of complex therapies within a defined clinical pathway, as well as patient surveys on various aspects of their disease. The integrated survey system for questionnaire-based data collection is equipped with a user interface specifically designed for pwMS. Currently, the Early Mobility Impairment Questionnaire (EMIQ) (62), the Multiple Sclerosis Walking Scale (MSWS-12), and Multiple Sclerosis Health Resource Survey (MS-HRS) (63, 64) are integrated in the questionnaire module. The medical staff manages the survey process (e.g., starting the survey) and provides assistance in answering questions. The mobile terminals are controlled by the *MSDS*^3*D*^ system located locally in the treatment center via a special server, which also regulates the data flow to and from the patient. Anonymity and data protection are guaranteed in a complex procedure with encrypted transmission. Patient surveys can thus be carried out digitally, as well as cognitive testing (Paced Auditory Serial Addition Test, Symbol Digit Modalities Test) and gait analysis (Timed 25-Foot Walk, 2 Min Walk Test), which have also been integrated into the system (65, 66).

### Connecting MSDS^3D^ to Other Data Infrastructures

The *MSDS*^3*D*^ infrastructure is also used for the European cohort of the Multiple Sclerosis Partners Advancing Technology and Health Solutions (MSPATHS) (67). This Biogen-funded global program for MS centers in Europe and North America successfully integrates digitally collected PROs into routine clinical care. Data collected via tablet includes general information about the person, health insurance, medical history of MS, use of medication and stimulants, laboratory results, vital signs, and MRI results. With the Multiple Sclerosis Performance Test (MSPT) (17, 18) in addition to the anamnestic parameters, all components of the MSFC as well as Neuro-QoL domains are recorded in a standardized manner, which can be visualized back to physician and patient using *MSDS*^3*D*^ (17, 18).

Various specific *MSDS*^3*D*^ modules allow standardized documentation and visualization of visit schedules and obligatory examinations using a vertical timeline that represents the examination times and horizontally arranged tasks with detailed parameters to be recorded. Administrative functions (e.g., creating a patient, registering a patient for an examination) and evaluation mechanisms are integrated into the patient management system via a toolbar. In diagnostic–therapeutic terms, the implemented instruments are based on the guidelines of the respective professional associations.

Further developments of *MSDS*^3*D*^ enable the web-based system-independent use of the platform and the integration of further participants in the treatment process. In addition, image and laboratory data relevant to MS can be captured in the *MSDS*^3*D*^ platform so that for the first time they can be systematically investigated combined with clinical data. By implementing lab data into the *MSDS*^3*D*^ transferred from the lab server, the analysis of laboratory data from the real world could be performed, easily linking clinical and laboratory data (68, 69).

### MSDS^3D^ as a Platform for Post-authorization Safety Studies

Particular emphasis was placed on the systematic collection of post-marketing safety data, as randomized controlled trials are not able to identify rare adverse events (70). This was recently shown in a systematic analysis of real-world studies for Fingolimod as an example (71). These post-authorization safety studies (PASS) are used to collect real-world data reflecting the real-life safety profile and utility of drugs, which is supported by *MSDS*^3^^D^ (72, 73).

For *MSDS*^3*D*^, drug-specific modules have been developed based on the proposed handling of the specific MS treatment (74, 75). The natalizumab module, specifically adapted for treatment with the monoclonal antibody natalizumab, contains all essential process components from the indication to the infusion procedure and the necessary control tests. The sequence of the visits and the instruments to be filled in are defined in the *MSDS*^3*D*^ natalizumab module. Subsequent instruments include disease history, EDSS, and MSFC as well as MRI and para-clinical parameters as lab data.

Specifically, a checklist was integrated that asks for the occurrence of common symptoms associated with progressive multifocal leukoencephalopathy (PML) as a possible side effect of natalizumab therapy and must be answered by each patient alone or in the presence of relatives before each infusion. This is also done via touch screen on the patient terminal or via touch pad. If the checklist contains warnings of a PML, so-called red flags appear, which require an immediate patient consultation with the attending physician. Once all the instruments necessary for the respective visit have been performed, the physician approves the infusion and only then can natalizumab be administered. The infusion itself is documented by the nurse who also arranges the next appointment using the MSDS^3D^ appointment manager. If the patient does not appear at the agreed appointment, the nurse and doctor are reminded by *MSDS*^3*D*^. If all instruments of therapy with natalizumab are marked green, the visit can be verified with the appropriate authorization and transferred to a central register (e.g., MS register or drug-specific register) in a pseudonymized manner. Compliance with the applicable national and European data protection regulations is guaranteed.

The findings from this pilot project are widely applied throughout Germany in the TRUST study initiated by Biogen to accompany patients under treatment with natalizumab (76). In addition, other modules have been developed to collect data of high-efficacy treatments with fingolimod (77, 78) and alemtuzumab (79). For alemtuzumab, *MSDS*^3*D*^ provides the necessary regular monitoring to ensure clinical vigilance after completion of the infusion courses over the necessary observation period of 4 years. It enables cross-sectoral standardized management and documentation of patients treated with alemtuzumab and can serve as a data entry system for various databases. We successfully linked clinical and imaging data of individual patients with the promising biomarker serum neurofilament light in Alemtuzumab-treated patients (80). For ocrelizumab, the CONFIDENCE study was integrated into the *MSDS*^3*D*^ platform as a large, non-interventional PASS that assesses long-term safety and effectiveness of Ocrelizumab and other MS treatments in comparison (81). Interestingly, these data will be integrated into other studies that have been developed to fulfill international regulatory requirements (EMA, FDA). Cladribine data are collected using the CLARION *MSDS*^3*D*^ module in Germany.

Additionally, *MSDS*^3*D*^ has found its way into the implementation of various scientific research projects as, for example, the multicenter study “Responsiveness of patient based outcome parameters in MS” (REPABO), in which pwMS were followed over up to 3 years and patients and their study physician rated different scales in parallel each year (82). A new physician tool, MSProDiscuss, was integrated in the PANGAEA module to facilitate physician–patient discussion in evaluating early, subtle signs of disease progression that represent the transition from relapsing–remitting to secondary progressive subtype (83).

## Perspective

In the age of large, complex, digitally available data sets (big data), and the establishment of suitable analytical methods, MS as a widespread chronic disease with various characteristics is predestined for large-scale data research approaches (84, 85). There are many prerequisites for finally investigating origin, progression modifiers, and chances of remission in greater depth with modern analytical methods in larger cohorts: the not yet completely clarified etiology, complex constellations of symptoms, the growing register landscape, as well as newly emerging markers and progression approaches (3, 33, 36). Big data analyses (e.g., data mining and machine learning) will not turn MS into a curable disease, but clear application goals can be derived:

- Comprehensive automated analysis of MRI data.
- Data-driven individualization of therapy recommendations.
- In real-time optimized follow-up by simultaneous consideration of numerous clinical outcomes and PROs.
- Combination of previously isolated domains such as genome, molecular, and epigenetic data.

Typical pitfalls of large complex data sets are potentially poor data quality, data inconsistency, poor data stability, securing patient protection and consent, and other legal barriers (13, 84, 86, 87). In addition, the interpretability of the results must be in the foreground when research moves away from the level of confirmatory hypothesis testing in order not to achieve irrelevant or misleading results. Ultimately, findings from big data have to be elaborated into new testable hypotheses. However, the data-oriented perspective also strengthens the view of the actual effect sizes (clinical important differences), where up to now all significant *p*-values of certified minimum effects have been too often classified as relevant.

Data from multiple sources such as registries, EMRs, and PASS can be separately analyzed and combined in a meta-analysis or brought together in a single big data source for MS research like the MS Data Alliance (13, 33). As we have described above, *MSDS*^3^^D^ has enabled us to free data sources, data collection systems, and study types from their pigeonholes in an integrative manner by building a system that already integrates data from registers, safety studies, and highly specialized EMR processes. Our vision is to provide a platform for holistic management of MS that allows parallel data collection for specific analysis.

Our next steps will be to include neurorehabilitation into this big data approach in MS by creating a neurorehabilitation module in MSDS^3D^. Here, too, we will follow the approach of making data collected as holistically as possible available to all participants in order to maintain multi-domain patient skills beyond isolated symptomatic approaches. On the professional side, the implementation of clinical pathways for the treatment of symptomatic disabilities will enable data-driven standardized care and make it measurable and verifiable (88). In our system, we have already implemented the necessary data to address, for example, motor deficits and psychosocial problems. We must now take these data and combine it with the efforts of doctors, nurses, and patients who already share and use parts of it. In this way, the electronically supported cycle of data from conception, collection, linking, and utilization can be completed.

## Author Contributions

TZ, RK, IV, and RH wrote the manuscript. All authors reviewed and approved the final manuscript.

## Conflict of Interest

TZ, IV, RH declare that the research was conducted in the absence of any commercial or financial relationships that could be construed as a potential conflict of interest. TZ received personal compensation from Biogen, Bayer, Celgene, Novartis, Roche, Sanofi, Teva for the consulting services and additional financial support for the research activities from Bayer, BAT; Biogen, Novartis, Teva, and Sanofi. RK is CEO of MedicalSyn GmbH. RH received personal compensation by Sanofi and travel grants by Celgene and Sanofi.
